# Editorial: *KRAS* in stage IV non-small cell lung cancer

**DOI:** 10.3389/fonc.2024.1517049

**Published:** 2025-03-10

**Authors:** Anneloes L. Noordhof, Torsten Gerriet Blum, Georgia Hardavella, Lizza E. L. Hendriks, Wouter H. van Geffen

**Affiliations:** ^1^ Department of Respiratory Medicine, Medical Center Leeuwarden, Leeuwarden, Netherlands; ^2^ Department of Pneumology, Lungenklinik Heckeshorn, Helios Klinikum Emil von Behring, Berlin, Germany; ^3^ Department of Internal Medicine/Pneumonology, Medical School Berlin, Berlin, Germany; ^4^ 6^th^ Department of Respiratory Medicine, “Sotiria” Athens’ Chest Diseases Hospital, Athens, Greece; ^5^ Department of Respiratory Medicine, GROW-Research Institute for Oncology and Reproduction, Maastricht University Medical Center, Maastricht, Netherlands

**Keywords:** non-small cell lung cancer, lung adenocarcinoma, Kirsten Rat Sarcoma (KRAS), KRAS G12C, KRAS inhibitors

Despite the continuous development of drugs targeting actionable genomic alterations (AGA’s), lung cancer remains the leading cause of cancer related death worldwide (1–3). In European populations, the most common AGA in stage IV non-small cell lung cancer (NSCLC), adenocarcinoma type, is the Kirsten Rat Sarcoma virus oncogene (*KRAS*) mutation (4, 5). *KRAS* was long thought to be “undruggable”, but for one of the *KRAS* subtypes, the G12C mutation, targeted therapy has become available which has significantly changed the therapeutic landscape for *KRAS* G12C mutated NSCLC. However, immunotherapy with immune checkpoint inhibitors (ICI), either alone or in combination with chemotherapy, is still the current first line of treatment for all *KRAS* subtypes including G12C (6, 7).

In the second line and beyond, G12C inhibitors such as sotorasib and adagrasib have been approved for patients with *KRAS* G12C mutated NSCLC. These are small molecules that bind into a specific groove in the G12C molecule to prevent downstream signalling and cell survival. Sotorasib and adagrasib both showed overall response rates of around 40% in the CodeBreaK 100 and KRYSTAL-1 phase II studies respectively (8, 9).

In the phase III CodeBreaK 200 trial, where sotorasib was compared to docetaxel, patients receiving sotorasib had a significant longer median progression free survival (PFS) compared to those treated with docetaxel (5.6 months vs. 4.5 months). However, overall survival (OS) was not different among treatment groups (10). In the KRYSTAL-12 phase III trial comparing adagrasib to docetaxel, adagrasib also showed a significantly improved median PFS (5.5 months for adagrasib vs. 3.8 months for docetaxel), but OS data has not been reported yet (11). In both phase III-trials, all patients were previously treated with platinum-based chemotherapy and ICI.

Nevertheless, despite these recent developments, optimizing the treatment strategy of *KRAS* mutated NSCLC remains subject of interest as the outcomes with sotorasib and adagrasib are still below those achieved with targeted therapies for several other AGA’s. The combined research efforts in this Research Topic in Frontiers of Oncology “*KRAS* in stage IV non-small cell lung cancer” cover multiple aspects of important challenges in treating *KRAS* mutated NSCLC.

The prognostic role of *KRAS* has been a subject of debate both in the past when only chemotherapy was available, and also in the current immunotherapy era (12, 13). Peng et al. explored the survival of 112 patients with *KRAS* mutated NSCLC in a Chinese study. Although there were no differences in PFS, patients treated with an ICI-based regiment (± chemotherapy) had a significantly better OS than those treated with chemotherapy alone. This effect was also reported in separate *KRAS* G12C and non-G12C cohorts as well as in a subgroup harbouring a *KRAS*/*TP53* co-mutation. This Chinese single centre study may have some limitations since it did not provide any information on the PD-L1 status, yet it offered an interesting insight into the survival of patients with a *KRAS* mutation in a non-Western cohort.

Notario et al. also aims to describe the clinical outcomes of 103 patients harbouring a *KRAS* mutation treated with ICI, mostly in first or second line, either as monotherapy or in combination with chemotherapy in a Spanish monocentric cohort. In this study, PD-L1 expression was higher among patients with the G12C subtype. Better OS and PFS were observed in patients with high PD-L1 expressing tumours, regardless of *KRAS* subtype mutation.

Although the focus in this Research Topic is on patients with stage IV disease, Eklund et al. published work from a different perspective. They describe the impact of the *KRAS* mutational status in patients with stage I-II NSCLC treated in a Swedish centre. The vast majority of these patients received surgical resection of their tumour. Of interest, although *KRAS* mutational status did not have a significant impact on OS, the authors reported a shorter OS in patients with a *KRAS* mutation: the mean (median not reached) OS was 63 months for patients with a *KRAS* mutation versus 74 months for patients without. The G12C mutation patients had a similar prognosis compared to those with non-G12C *KRAS* mutations. Since there were only 113 patients with a *KRAS* mutation in this study, larger studies are needed to establish the prognostic role of *KRAS* in this patient category.

*KRAS* is a heterogeneous disease, where multiple co-mutations can co-occur and may affect clinical outcomes. On this ground, Frille et al. advocate for extensive predictive testing including broad panels for mutation analysis to better estimate the prognosis and treatment options for patients with advanced NSCLC. They present the survival of more than 4000 patients with different mutations: *KRAS*, *STK11*, *KEAP1* and *TP53*, either alone or in complex combinations. Patients with a *KRAS*-only mutation, or with a combination of *KRAS* + *STK11* had the longest OS. The *TP53*-comutation showed a negative influence on *KRAS* mutated NSCLC, as in this group the OS was significantly reduced by more than 30%.

The narrative review of Sreter et al. offers a detailed overview regarding the molecular basis, the role of co-mutations and an overview of clinical evidence for *KRAS* inhibition with sotorasib and adagrasib. Moreover, they offer a review of literature of intracranial responses with these two G12C-inhibitors, and they propose mechanisms of acquired resistance to G12C-inhibitors and future strategies to overcome them.

Chour et el. provide a review in which they summarize the *KRAS* pathway and the mechanism of sotorasib and adagrasib, that both bind to the inactive GDP-bound state of *KRAS*. The also discuss mechanism of resistance to these G12C-inhibitors, either primary resistance, i.e. new co-occurring mutations that prevent the binding of inhibitors, or acquired resistance, for example gain-of-function mutations in other oncogenes and thereby bypassing *KRAS*.

Current guidelines advise testing for PD-L1 status and molecular testing for AGA in patients with metastatic NSCLC (6). However, obtaining histology or cytology samples can be challenging. Cai et al. preformed a systematic review and meta-analysis to investigate the diagnostic accuracy of *KRAS* detection in plasma samples. Plasma NGS could be a suitable alternative when tissue samples are not available as it detects *KRAS* with high accuracy.

Since G12C inhibitors are now available in the second line treatment setting and beyond, managing toxicity and optimal dosing of sotorasib remains a challenge. Shigaki et al. explored the possible relation between blood sotorasib levels and therapeutic outcomes and adverse event in five patients treated with sotorasib but found no association. Nevertheless, this is an interesting concept for further evaluation in a larger number of patients and it could possibly offer insights into more personalized dosing strategies in the future.

The combined efforts of these studies published in this Research Topic have contributed in decreasing the knowledge gap on how to optimize treatment strategies for patients with *KRAS* mutated NSCLC. The treatment landscape is anticipated to change further with the development of inhibitors of other *KRAS* mutational subtypes and pan-*KRAS* inhibitors (14). Ongoing research in the acquired resistance to G12-inhibitors and their administration in combination with other types of therapy could further change the treatment landscape, but additional research is needed in these areas.
